# Systemic lupus erythematosus complicated by retroperitoneal fibrosis: A case report and literature review

**DOI:** 10.1097/MD.0000000000041208

**Published:** 2025-01-03

**Authors:** Song Cheng, Sikai Wu, Neng Wang, Wenjie Xu, Fuquan Wei, Weiqun Ao, Li Yuan, Xiaoxiang Ning, Yichuan Mao, Xianzhen Zhang, Guoqun Mao

**Affiliations:** a Department of Radiology, Tongde Hospital of Zhejiang Province, Hangzhou, Zhejiang Province, China; b Zhejiang Chinese Medical University, Hangzhou, Zhejiang Province, China.

**Keywords:** glucocorticoid, magnetic resonance imaging, retroperitoneal fibrosis, systemic lupus erythematosus

## Abstract

**Rationale:**

Systemic lupus erythematosus (SLE) is a chronic autoimmune disease that affects multiple organ systems. Retroperitoneal fibrosis (RPF) is a rare condition characterized by the development of fibrous tissue in the retroperitoneal space. The coexistence of SLE and RPF is extremely uncommon, and this report aims to enhance understanding of this complex relationship.

**Patient concerns:**

A 32-year-old woman presented with sudden-onset syncope. Her medical history revealed a 5-year history of SLE, and imaging studies identified a retroperitoneal mass.

**Diagnoses:**

A comprehensive diagnostic workup, including magnetic resonance imaging (MRI) and biopsy, confirmed retroperitoneal fibrosis secondary to SLE.

**Interventions:**

The patient was treated with high-dose corticosteroids, immunosuppressive therapy, and the biologic agent rituximab.

**Outcomes:**

The patient’s symptoms markedly improved, and follow-up MRI demonstrated significant regression of the retroperitoneal lesion.

**Lessons:**

RPF associated with SLE is exceptionally rare. This case underscores the importance of early diagnosis and a coordinated multidisciplinary approach in managing such complex conditions. Glucocorticoid therapy remains the cornerstone of treatment, augmented by immunosuppressants and biologic agents when necessary.

## 1. Introduction

Systemic lupus erythematosus (SLE) is a classic diffuse connective tissue disease characterized by autoimmune inflammation, often affecting multiple organ systems including the skin, mucous membranes, musculoskeletal system, urinary system, endocrine system, circulatory system, respiratory system, digestive system, and nervous system. It is typically associated with immunological abnormalities, particularly autoantibody positivity.^[1]^ The disease typically alternates between periods of remission and recurrence, and in severe cases, it can lead to organ dysfunction, organ failure, or even death. Retroperitoneal fibrosis (RPF) is a rare condition characterized by chronic inflammation and significant fibrosis of retroperitoneal tissue, often involving the abdominal aorta, its branches, ureters, or other abdominal organs.^[2]^ SLE and RPF are 2 distinct diseases with different pathogenesis, but their overlap presents unique diagnostic and therapeutic challenges. Both conditions can manifest with nonspecific symptoms such as abdominal or flank pain, fatigue, and weight loss, which can make early diagnosis challenging. Additionally, the co-occurrence of SLE and RPF can obscure typical clinical patterns, leading to delays in recognition and treatment.

SLE is relatively rare, and cases complicated by RPF are even rarer. This overlap underscores the importance of considering secondary causes of RPF, particularly in patients with systemic autoimmune diseases like SLE. Recognizing the imaging features and clinical clues of these overlapping conditions is crucial for timely diagnosis and effective management. In this paper, we describe the imaging manifestations and treatment of a young female patient with SLE complicated by RPF.

## 2. Case description and ethics approval

Approval for this case report was obtained from the Ethics Committee of Tongde Hospital of Zhejiang Province. Written informed consent was also obtained from the patient for the publication of her medical information.

### 2.1. Case background

On June 13, 2022, a 32-year-old woman was admitted to the hospital following a sudden episode of syncope. She had a 10-year history of Hashimoto thyroiditis, managed with levothyroxine replacement therapy.

### 2.2. Medical history

Three years prior: the patient was hospitalized at a local facility for a rash and lower limb edema. A renal biopsy confirmed SLE with class IV lupus nephritis. She was treated with glucocorticoids and cyclophosphamide (CTX) for 6 months, which alleviated her symptoms. However, persistent mild proteinuria and hypoalbuminemia necessitated the addition of tacrolimus.

**Figure 1.**
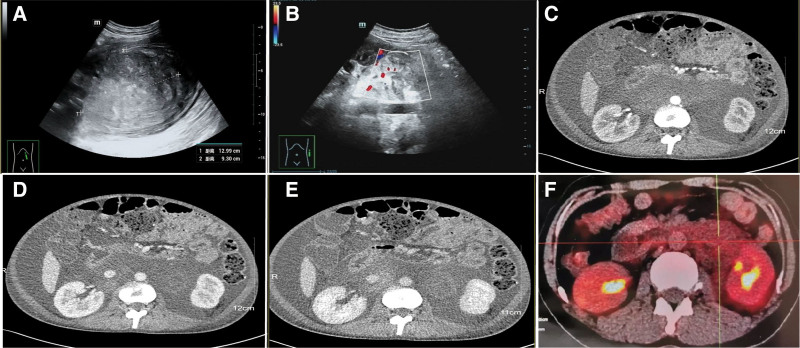
Imaging features of a 32-year-old woman with a retroperitoneal mass (A and B) Ultrasound revealed multiple hypoechoic nodules in the retroperitoneal area, with indistinct borders and an irregular echo pattern. CDFI showed minimal blood flow, suggesting a fibrotic nature of the mass. (C–E) CT scan demonstrated a retroperitoneal mass with a shadow. The 3-phase enhancement did not show any significant increase in intensity, indicating a nonmalignant lesion. (F) PET-CT showed patchy hyperdensity in the retroperitoneal space, with slightly increased FDG metabolism diffusely, suggesting inflammation or fibrosis rather than malignancy. Clinical significance: The imaging features, including ultrasound, CT, and PET-CT findings, assist in differentiating RPF from other retroperitoneal pathologies, such as malignancies. The absence of significant enhancement on CT and the lack of increased FDG uptake on PET-CT support a diagnosis of RPF rather than cancer. CDFI = Color Doppler flow imaging, CT = computer tomography, PET-CT = positron emission tomography-computed tomography, RPF = retroperitoneal fibrosis.

**Figure 2.**
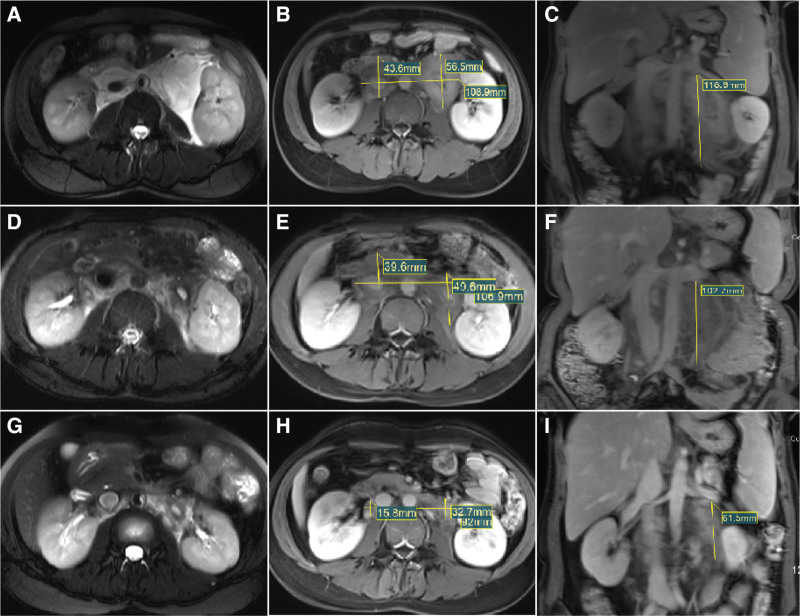
MRI images of a 32-year-old female patient before and after treatment. (A–C) MRI images from 1 year before admission. (A) Patchy hyperintensity in the retroperitoneal area on T2-weighted imaging. (B and C) Slight enhancement of the lesion and its extent on T1-weighted imaging. (D–F) MRI images after the first treatment. (D) Reduced T2 hyperintensity in the retroperitoneal patch, indicating improvement after treatment. (E and F) Significant reduction in the lesion area, reflecting a positive response to treatment. (G and I) Follow-up MRI images. (G) Decreased T2 signal intensity in the retroperitoneal area, indicating reduced inflammation. (H and I) Significant reduction in the lesion area, demonstrating the effectiveness of the treatment. Clinical Significance: MRI monitoring of retroperitoneal fibrosis shows a significant reduction in both the lesion area and T2 signal intensity after treatment, indicating a favorable treatment response and disease improvement. MRI = magnetic resonance imaging.

One year prior: the patient presented to our hospital with transient lumbar soreness. Imaging studies revealed a retroperitoneal mass (ultrasound: Fig. 1A and B; magnetic resonance imaging (MRI): Fig. 2A–C). The mass demonstrated delayed enhancement, suggestive of RPF or lymphoma. PET-CT indicated mildly increased metabolic activity (Fig. 1F). The patient declined further evaluation and hospitalization.

From January to March 2022: To prepare for pregnancy, she discontinued glucocorticoids and CTX and switched to traditional Chinese medicine (details unknown). During this period, she did not undergo regular monitoring.

### 2.3. Hospital course

**Figure 3.**
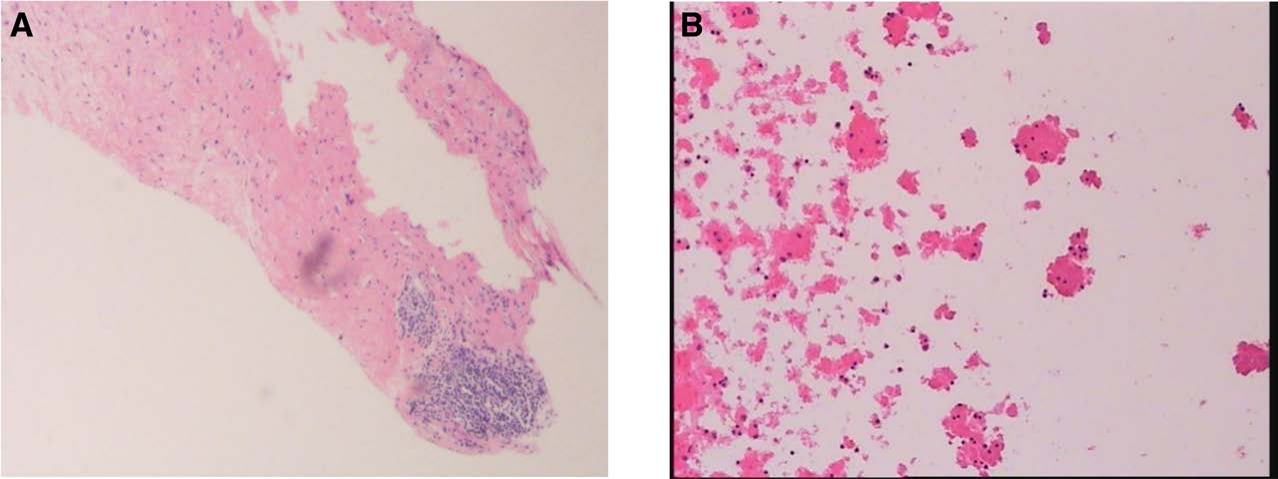
Pathological image of a retroperitoneal space occupied puncture. The tissue section revealed hyperplasia of fibrous tissue, a small area of lymphocyte infiltration, and no evidence of plasma cell infiltration. Immunohistochemical staining: A2: pan-cytokeratin(–), Vimentin(+), smooth muscle actin(–), S-100 protein(–), SRY-box transcription factor 10(–), β-Catenin(–), immunoglobulin G(–), immunoglobulin G4(–), cluster of differentiation 38(–), Ki-67 proliferation index(+,<1%), leukocyte common antigen(+), Desmin(+), Myoglobin(–). A diagnosis of retroperitoneal fibrosis was made based on immunohistochemical findings and the patient’s clinical history.

On June 13, 2022, the patient was readmitted to the emergency department after another episode of syncope. Laboratory tests revealed severe pancytopenia, hypoalbuminemia, and active lupus (immunological findings are detailed in Table 1). Imaging studies showed a significant increase in the size of the retroperitoneal mass, accompanied by massive ascites (enhanced CT: Figs. 1C–E).An ultrasound-guided biopsy confirmed RPF, with negative immunohistochemical staining for IgG and IgG4 (Fig. 3A and B).

**Table 1:** Laboratory findings at admission (June 2022).

Parameter	Result	Normal range	Remarks
Hemoglobin	49 g/L	120 to 160 g/L	Severe anemia
Platelet count	1 × 10^9^/L	100 to 300 × 10^9^/L	Pancytopenia
Albumin	22.1 g/L	35 to 50 g/L	Hypoalbuminemia
ANA	1:320	Negative	
Anti-dsDNA antibody	85.7 IU/mL	0 to 30 IU/mL	Strongly positive
Complement C3	0.15 g/L	0.9 to 1.8 g/L	Markedly decreased
Complement C4	0.02 g/L	0.1 to 0.4 g/L	Markedly decreased
IgG	26.80 g/L	7 to 16 g/L	Markedly elevated

This table presents the patient’s laboratory results upon admission, compared to the normal reference ranges. The results show severe anemia, pancytopenia (including thrombocytopenia), and hypoalbuminemia. Additionally, ANA and anti-dsDNA antibodies were strongly positive, indicating active SLE. Complement C3 and C4 levels were markedly decreased, reflecting heightened immune system activity and inflammation. IgG levels were significantly elevated, suggesting abnormal immune activation.

Abbreviations: ANA = antinuclear antibody, Anti-dsDNA = anti-double-stranded DNA, IgG = immunoglobulin G, SLE = systemic lupus erythematosus.

### 2.4. Treatment and follow-up

During hospitalization: the patient received high-dose methylprednisolone pulse therapy (500 mg/d for 3 days), followed by a gradual taper. She was also treated with intravenous immunoglobulin (20 g/d), rituximab (500 mg, single dose), and supportive care. Laboratory results showed gradual improvement in hematologic parameters and complement levels (Table 2). MRI revealed a reduction in the size of the retroperitoneal lesion (Table 3 and Fig. 2D–F).

**Table 2:** Key laboratory indicators before and after treatment.

Indicator	At admission (June 2022)	After 10 mo (April 2023)	Change
Hemoglobin	49 g/L	120 g/L	Restored to normal range
Platelet count	1 × 10^9^/L	150 × 10^9^/L	Significant improvement
Complement C3	0.15 g/L	0.69 g/L	Partial recovery
Complement C4	0.02 g/L	0.32 g/L	Partial recovery
24-h urinary protein	7.6 g	280 mg	Restored to normal range
IgG	26.80 g/L	14.2 g/L	Restored to normal range

This table presents the key laboratory indicators at 2 points: admission (June 2022) and after 10 mo of treatment (April 2023). Significant improvements were observed in several parameters, indicating a positive therapeutic response. Hemoglobin levels and platelet count returned to normal ranges, while complement levels (C3 and C4) partially recovered. The 24-h urinary protein level also significantly decreased, returning to the normal range. Additionally, IgG levels were restored to normal levels, reflecting overall improvement in the patient’s condition.

Abbreviation: IgG = Immunoglobulin G.

**Table 3:** Imaging changes during follow-up.

Time	Imaging findings	Remarks
August 2021	Retroperitoneal mass (10.1 × 7.5 cm), T1 signal hyperintensity, increased T2 signal intensity	MRI suggested RPF or lymphoma
June 2022	Mass enlarged to 15.0 × 9.1 cm, with ascites and bowel wall edema	Disease progression
April 2023	Mass reduced to 6.0 × 3.5 cm, with significantly diminished signal intensity	Positive response to treatment

This table summarizes the changes observed in imaging studies during the patient’s follow-up period, showing initial disease progression followed by a positive response to treatment, as evidenced by a reduction in mass size and improved imaging characteristics.

Abbreviation: MRI = magnetic resonance imaging.

### 2.5. Over the 10-month follow-up period

Medication adjustments: the patient continued treatment with low-dose glucocorticoids, tacrolimus (1 mg twice daily), and belimumab (600 mg). Glucocorticoid doses were gradually tapered to the minimal effective dose.

Treatment response: The retroperitoneal lesion decreased significantly in size. Proteinuria resolved (24-hour urinary protein decreased from 7.6 g to 280 mg), and serum albumin and immunological markers progressively normalized (Table 3 and Fig. 2G–I).

Infection management: during follow-up, the patient experienced upper respiratory tract infections, pneumonia, and herpes zoster, all of which were promptly treated with favorable outcomes.

## 3. Discussion

RPF, first described by Ormond in 1948, is characterized by the proliferation of fibrous tissue in the retroperitoneal space.^[3]^ RPF can be secondary, often associated with malignancies, or idiopathic retroperitoneal fibrosis (IRF), frequently linked to autoimmune diseases such as SLE, autoimmune thyroiditis, and others.^[4–6]^ Although many IRF cases are associated with IgG4-related disease (IgG4RD), not all are; some are classified as “IgG4-independent.”^[7]^

In this case, the patient was diagnosed with SLE-related RPF rather than IgG4RD. Despite low IgG4 levels (<0.400 g/L), pathological evaluation revealed no significant increase in IgG4, effectively excluding IgG4RD. Notably, IgG4RD does not always present with elevated serum IgG4 levels, emphasizing the need for pathological confirmation.^[8]^

SLE-associated RPF is extremely rare, with only a few cases reported since the first in 1966.^[9–15]^ SLE and RPF may be linked through various pathophysiological mechanisms. As an autoimmune disease, SLE is characterized by chronic systemic inflammation that promotes fibrotic processes. Pro-inflammatory cytokines (e.g., IL-6 and TNF-α) and chemokines (e.g., CCL2 and CXCL12) activate fibroblasts, leading to excessive collagen deposition and driving fibrosis.^[4]^ Additionally, SLE-associated vasculitis and tissue hypoxia may exacerbate fibrosis through the activation of hypoxia-inducible factor-1α (HIF-1α) and inhibition of local angiogenesis.^[16]^ Autoantibodies in SLE, such as anti-double-stranded DNA antibodies, may cross-react with local antigens. This interaction can induce immune complex deposition, complement activation, and inflammation, further aggravating tissue damage.^[17,18]^ Abnormal immune cell infiltration and activation also play a critical role in the pathology of RPF. For example, defects in regulatory T cells (Tregs) and an imbalance in helper T cell subsets (Th1/Th17) can sustain chronic immune responses.^[18]^ Moreover, profibrotic factors such as transforming growth factor-β (TGF-β) and connective tissue growth factor synergistically activate signaling pathways, further accelerating the fibrotic process.^[19]^ These mechanisms suggest that SLE-associated RPF results from the interplay between immune system dysregulation and fibrotic responses. This highlights the importance of comprehensive immunological and pathological evaluations to improve diagnostic accuracy and guide effective treatment.

Patients with SLE-associated RPF often present with systemic symptoms of SLE and specific features of retroperitoneal fibrosis. These overlapping characteristics can complicate diagnosis and treatment, potentially leading to misdiagnosis or delayed diagnosis. In this case, the primary differential diagnoses included IgG4-related disease (IgG4RD), malignancies, and infectious diseases. IgG4RD is 1 of the most common causes of RPF, typically characterized by elevated serum IgG4 levels and pathological infiltration of IgG4-positive plasma cells. However, in this patient, serum IgG4 levels were low (<0.400 g/L), and pathological examination showed no significant increase in IgG4-positive cells, effectively excluding IgG4RD. Fibrotic lesions in the retroperitoneal region may also result from malignant lymphoma or metastatic tumors. FDG PET/CT is instrumental in differentiating these conditions. In this case, PET/CT revealed diffuse metabolic activity rather than focal hyperactivity, which is inconsistent with malignancies. Furthermore, pathological biopsy confirmed the absence of malignant cells. Certain chronic infections, such as tuberculosis or fungal infections, can mimic RPF-like lesions. However, this patient lacked clinical and laboratory evidence of infection, such as fever or positive blood cultures, supporting a noninfectious etiology. Diagnosing SLE-associated RPF relies on clinical presentation, laboratory tests, imaging studies, and biopsy findings. Table 4 summarizes the clinical and laboratory findings from previously reported cases and this patient.

**Table 4:** General clinical information and main laboratory tests of each case.

References	Gender	Age (yr)	Clinical manifestation	Main laboratory indicators
Lipman et al^[9]^	Female	29	Hematuria, edema, hepatosplenomegaly	BUN 78 mg/100 cc, urine protein 1.3g/d
Lloyd et al^[10]^	Female	17	Butterfly erythema on face, hepatosplenomegaly, convulsion	ESR 126 mm/h, BUN 12 mg/100 mL
Uchino et al^[11]^	Female	21	Dysentery, abdominal pain, vomiting, weight loss and frequent urination	Anti-double-stranded DNA antibody: 23 IU/mL, serum complement: 16.8 u/mL, antinuclear antibody: 640 times, urine protein: 5.2g/d
García-Morteo et al^[12]^	Male	26	Photosensitive cheek rash, diffuse alopecia, edema of limbs	ESR 42 mm/h, anti-double-stranded DNA antibody positive, serum C3: 25 mg/dL, C4: 8 mg/dL
Bashour et al^[13]^	Female	17	Intermittent pain in low back and left lower abdomen	ESR 55 mm/h, direct Coombs test positive, antinuclear antibody strongly positive, anti-double-stranded DNA antibody strongly positive
Okada et al^[14]^	Female	54	Anorexia and nausea, pretibial edema and weight gain	Antinuclear antibody: 160 times, anti-double stranded DNA: 25.9 IU/mL, anticardiolipin IgG: 13 u/mL
Soyuöz et al^[15]^	Male	28	Abdominal pain, nausea, maculopapules on limbs, history of deep venous thrombosis	ESR 40 mm/h, CRP: 8.8 mg/dL, Fibrinogen: 712 mg/dL, antinuclear antibody: 320 times, anticardiolipin IgG: >300 GPL/mL, antiphospholipid IgG: 232 GPL/mL
Our case	Female	32	Sudden syncope and ascites, history of SLE	ESR: 46 mm/h, High-sensitivity C-reactive protein (hs-CRP): 1.2 mg/L, antinuclear antibody (ANA): 1:100, anti-double-stranded DNA antibody (Anti-dsDNA): 85.7 IU/mL, Thyroid peroxidase antibody (TPOAb): 809.60 IU/mL, thyroglobulin antibody (TgAb): >2481.00 IU/mL

This table provides a concise summary of each case’s gender, age, clinical manifestations, and key laboratory findings, allowing for a quick comparison across cases.

Imaging studies play a vital role in assessing the extent and characteristics of RPF. Ultrasound revealed hypoechoic nodules in the retroperitoneal area with reduced blood flow, providing an initial clue for RPF. CT scans clearly delineated the extent of the lesion and its anatomical relationship with surrounding tissues but offered limited insights into tissue composition. MRI, with its superior resolution, is particularly useful for evaluating disease extent and activity.^[20]^ In this case, MRI demonstrated low signal intensity on T1-weighted images and high signal intensity on T2-weighted images, especially during the acute phase or early stages. diffusion-weighted imaging indicated mild diffusion restriction, while enhanced T1-weighted images showed slight enhancement. These features supported the diagnosis of RPF and helped differentiate it from malignant lymphadenopathy.^[21,22]^

Treatment for RPF typically includes glucocorticoids, immunosuppressants, and biological agents. In this case, the treatment regimen included glucocorticoids, tacrolimus, and belimumab. Belimumab, a biologic agent targeting the B-cell activating factor, has shown efficacy in treating refractory SLE and RPF. Biological agents such as belimumab, rituximab, and tocilizumab play significant roles in treating RPF, particularly in cases resistant to conventional therapies. These biologics target specific immune pathways to reduce fibrosis and inflammation, thus improving patient outcomes.

In cases with complications such as severe hydronephrosis, surgical intervention may be necessary. However, in this case, the patient’s SLE was active, and RPF did not cause organ dysfunction such as ureteral obstruction or hydronephrosis; thus, pharmacological therapy remained the primary treatment. Monitoring RPF patients typically involves assessing subjective symptoms, regularly measuring ESR and CRP levels, and using imaging studies to evaluate disease progression or treatment response.^[22]^ Table 5 summarizes reported cases, along with pathological biopsy and key imaging findings of this case.

**Table 5:** Histological biopsy results, imaging manifestations and treatment plans of each case.

References	Renal biopsy	Retroperitoneal mass biopsy	Imaging manifestation	Treatment plans
Lipman et al^[9]^	Lupus glomerulonephritis	Vascular fibroadipose tissue	IVP: normal kidneys, poor overlap at left pelvic and ureteral junction	Nephrostomy, glucocorticoids 60 mg/d
Lloyd et al^[10]^	Lupus glomerulonephritis	–	IVP: bilateral hydronephrosis, ureteral narrowing above the bladder	Ureteral intubation, glucocorticoids 60 mg/d
Uchino et al^[11]^	–	Fibrous tissue around ureter/bladder	IVP: significant hydronephrosis, ureteral stenosis; CT: bilateral ureteral adhesions	Deceased
García-Morteo et al^[12]^	Diffuse proliferative glomerulonephritisritis	Sclerosis, lymphocyte proliferation	IVP: normal.	Glucocorticoids 60 mg/d
Bashour et al^[13]^	Membranous nephropathy	Fibrous tissue, no malignancy	Ultrasound: enlarged kidneys, retroperitoneal mass; CT: periaortic adenopathy, pelvic mass	Glucocorticoids 80 mg/d
Okada et al^[14]^	–	–	MRI: T1 hypointense, T2 hyperintense area around aortic bifurcation, bilateral hydronephrosis	Double J-tube, glucocorticoids 40 mg/d
Soyuöz et al^[15]^	–	–	CT: thoracoabdominal aorta wall thickening, increased adipose tissue density	Glucocorticoids, cyclophosphamide, MMF
Our case	Lupus glomerulonephritis	Fibrous tissue, mild lymphocyte infiltration	Ultrasound: hypoechoic nodules; CT: retroperitoneal mass, no enhancement; MRI: patchy high T2 signal, mild diffusion; PET-CT: diffuse FDG uptake	Glucocorticoids, tacrolimus, belimumab

This table provides a concise overview of the key findings and treatments for each case, with a focus on the renal biopsy, retroperitoneal biopsy, imaging results, and treatment plans.

Abbreviations: CT = computed tomography, IVP = intravenous pyelogram, MRI = magnetic resonance imaging, PET-CT = positron emission tomography-computed tomography.

## 4. Conclusion

This case highlights the importance of comprehensive diagnostic evaluation and individualized multidisciplinary treatment approaches. Few documented cases report the concurrent occurrence of SLE and RPF. SLE, a connective tissue disease characterized by chronic systemic inflammation, is a significant contributing factor to the development of RPF. Glucocorticoids remain the primary treatment modality, often supplemented with immunosuppressants or biologic agents. In cases resistant to conventional therapies, biologic agents such as belimumab, rituximab, or tocilizumab should be considered, as they target specific immune pathways to reduce fibrosis and inflammation. Ureterolysis is a viable therapeutic option for patients with hydronephrosis caused by ureteral obstruction. Moreover, MRI is a valuable tool for assessing pathological states and evaluating therapeutic efficacy. Regular follow-up with imaging and laboratory tests is essential to monitor disease progression and treatment response. Further research is needed to elucidate the pathogenesis and determine optimal management strategies for co-occurring autoimmune and fibrotic diseases.

## Author contributions

**Conceptualization:** Song Cheng, Sikai Wu, Weiqun Ao, Fuquan Wei, Xiaoxiang Ning, Yichuan Mao.

**Data curation:** Neng Wang, Li Yuan.

**Investigation:** Wenjie Xu.

**Writing – original draft:** Song Cheng.

**Writing – review & editing:** Xianzhen Zhang, Guoqun Mao.
